# Dexamethasone Preconditioning Improves the Response of Collagen-Induced Arthritis to Treatment with Short-Term Lipopolysaccharide-Stimulated Collagen-Loaded Dendritic Cells

**DOI:** 10.1155/2013/296031

**Published:** 2013-05-29

**Authors:** Corina Peña, David Gárate, Juan Contreras-Levicoy, Octavio Aravena, Diego Catalán, Juan C. Aguillón

**Affiliations:** ^1^Immune Regulation and Tolerance Research Group, Programa Disciplinario de Inmunología, Instituto de Ciencias Biomédicas (ICBM), Facultad de Medicina, Universidad de Chile, Avenida Independencia 1027, Santiago, Chile; ^2^Millennium Institute on Immunology and Immunotherapy-Chile (P09-016-F), Santiago, Chile

## Abstract

*Background*. Pharmacologically modulated dendritic cells (DCs) have been shown to restore tolerance in type II collagen-(CII-) induced arthritis (CIA). We examined the effect of dexamethasone (DXM) administration as a preconditioning agent, followed by an injection of lipopolysaccharide-(LPS-) stimulated and CII-loaded DCs on the CIA course. *Methods*. After CIA induction, mice pretreated with DXM were injected with 4-hour LPS-stimulated DCs loaded with CII (DXM/4hLPS/CII/DCs). *Results*. Mice injected with DXM/4hLPS/CII/DCs displayed significantly less severe clinical disease compared to animals receiving 4hLPS/CII/DCs alone or those in which only DXM was administered. Cytokine profile evaluation showed that CD4+ T cells from DXM/4hLPS/CII/DCs and 4hLPS/CII/DCs groups release higher IL-10 levels than those from mice receiving DXM alone or CIA mice. CD4+ T cells from all DC-treated groups showed less IL-17 release when compared to the CIA group. On the contrary, CD4+ T cells from DXM/4hLPS/CII/DCs and 4hLPS/CII/DCs groups released higher IFN-**γ** levels than those from CIA group. *Conclusion*. A combined treatment, including DXM preconditioning followed by an inoculation of short-term LPS-stimulated CII-loaded DCs, provides an improved strategy for attenuating CIA severity. Our results suggest that this benefit is driven by a modulation in the cytokine profile secreted by CD4+ T cells.

## 1. Introduction

Current evidence indicates that dendritic cells (DCs) functions are related to the stage of maturation and associated cytokine production profile. Unlike mature DCs, immature and semimature DCs exhibit a reduced costimulatory capacity and show a distinctive IL-10 high/IL-12 low cytokine profile, which might endow them with tolerogenic functions [1]. In order to induce and maintain a tolerogenic phenotype on *in vitro *generated DCs for therapeutic purposes, several strategies have been used, including modulation with agents such as IL-10, TNF, neuropeptides, lipopolysaccharide (LPS), dexamethasone (DXM), and vitamin D3 plus LPS, among others [2–7]. Our group has demonstrated that short-term LPS stimulation can induce IL-10-producing tolerogenic DCs that, when administered to mice with established collagen-induced arthritis (CIA), can interfere with the disease course [6, 8]. 

A major challenge that therapeutic administration of tolerogenic DCs has to face is to avoid the conversion of DCs into immunogenic antigen-presenting cells as they encounter a proinflammatory environment. This goal could be achieved if a transient immunosuppression is induced in the recipient just before receiving an injection with tolerogenic DCs. Data from animal models of autoimmunity and patients affected by autoimmune diseases has shown that nonmyeloablative immunosuppression followed by hematopoietic stem cell transplantation led to a reset of the dysregulated immune system of the recipient, which in some cases, reach complete remission of the disease [9]. 

In the present study, we demonstrate that the outcome of CIA mice receiving short-term LPS-stimulated collagen-loaded DCs inoculation can be improved by a previous DXM administration. In addition, we prove that CD4+ T cells from mice receiving the combined treatment produced high levels of IL-10 and IFN-*γ*, while they were low IL-17 producers.

## 2. Materials and Methods

### 2.1. Bone Marrow-Derived DCs

Bone marrow cells from DBA1/lacJ mice were differentiated into DCs as described by Salazar et al., 2008 [6]. After 6 days of culture, bone marrow-derived DCs were purified by positive selection using magnetic beads coupled to anti-CD11c antibodies (Miltenyi Biotec, Germany) and then stimulated with 1 *μ*g/mL LPS (*Escherichia coli*, Sigma-Aldrich, USA) for the last 4 hours (4hLPS/CII/DCs) and loaded for 24 hours with bovine type II collagen (CII) (Chondrex, Redmond, WA, USA), or left unloaded (4hLPS/DCs). LPS-untreated DCs loaded with CII (0hLPS/CII/DCs) or unloaded (0hLPS/DCs) or treated for 24 hours with LPS (24hLPS/DCs) were used as controls.

### 2.2. Characterization of DCs Phenotype and Cytokine Secretion Profile

The following antibodies were purchased from eBioscience (San Diego, CA, USA): fluorescein isothiocyanate-(FITC-) labeled anti-CD11c, phycoerythrin-(PE-) labeled anti-CD86, anti-MHC class II, and anti-CD40. Stained DCs were acquired in a FACSCalibur flow cytometer (BD Bioscience, San Diego, CA, USA) and data was analyzed using the WinMDI 2.9 software. IL-10 and IL-12 production was measured in supernatants from differentially treated DCs by ELISA (Bender MedSystems, Austria).

### 2.3. CIA Induction and Clinical Evaluation

CIA induction and clinical evaluation with the Joint Score and the Swollen Joint Severity Score were performed as described by Salazar et al., 2008 [6]. Protocols were approved by the Bioethics Committee of Universidad de Chile.

### 2.4. DXM Administration

DXM (Sigma-Aldrich, USA) was intraperitoneally administered in different doses (0.5, 1.0, and 2.0 mg/kg). The drug was given daily for 6 days, starting at day 29 until day 34 after disease induction.

### 2.5. DCs Inoculation

Differentially treated DCs (5 × 10^5^) were intraperitoneally administered to 8–10 mice per group by a single injection at day 35 after CIA induction.

### 2.6. CD4+ T Cells Cytokines Production Assessment

 Spleens were obtained from different groups of mice at day 47 after first CII inoculation. CD4+ T cells from spleens were purified by negative selection using magnetic beads (Miltenyi Biotec). Isolated CD4+ T cells were incubated in the presence of 5 *μ*g/mL concanavalin A at 37°C for 72 hours, and supernatants were collected and frozen at −85°C. IL-10, IFN-*γ*, and IL-17 were quantified by ELISA (Bender MedSystems). 

### 2.7. Statistical Analysis

 Comparisons between different groups of DCs or CD4+ T cells were performed with a one-way ANOVA test, corrected with Bonferroni's post-test. Two-way ANOVA test, corrected with Bonferroni's post-test, was applied for comparisons between clinical scores of different groups of mice. *P* < 0.05 was considered statistically significant. For statistical analyses and graphics, the software GraphPad Prism 4 was used.

## 3. Results

### 3.1. DXM Dosing for CIA Inhibition

**Figure 1:**
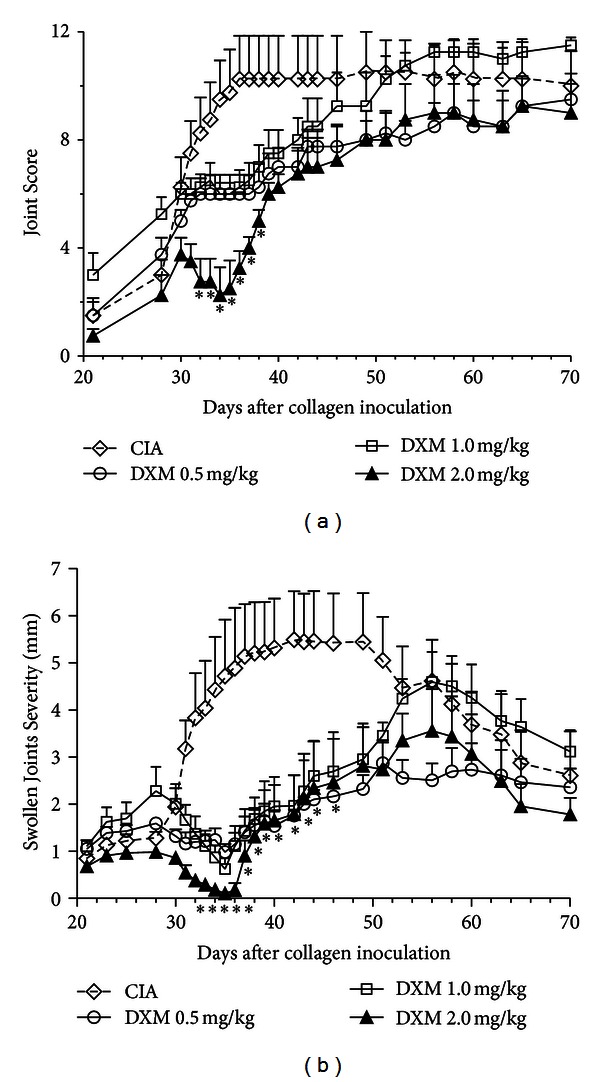
Dexamethasone (DXM) dosing for collagen-induced arthritis (CIA) inhibition. (a) Different doses of DXM (0.5, 1.0, and 2.0 mg/kg) were intraperitoneally administered to DBA1/lacJ mice from days 29 to day 34 after CIA induction. The mean Joint Score and the Swollen Joint Severity Score were determined until day 70. Data from a representative experiment of three experiments performed with 8–10 mice per group.*P < 0.001, in DXM 2.0 mg/kg versus CIA group.

 In order to define the dose of DXM capable of providing the most intensive anti-inflammatory effect on CIA mice, three groups of animals were treated with 0.5, 1.0, and 2.0 mg/kg DXM, respectively, for six consecutive days, starting at day 29 after the first CII inoculation. As depicted in Figure 1, all DXM doses were able to interfere with the onset and the course of CIA. However, from day 39 and 46, respectively, both Joint Score and Swollen Joint Severity Score increased irrespectively of the DXM dose administered, reaching values that did not differ statistically from those observed in untreated CIA animals. According to the Joint Score, mice that received DXM at 2.0 mg/kg, were the only group to show significant lower mean scores compared with CIA mice up to day 39 (Figure 1). For this reason, we used this dose in subsequent experiments.

### 3.2. Effect of DXM Pretreatment Followed by Short-Term LPS-Stimulated DCs Inoculation on Established CIA

 To assess whether the transient anti-inflammatory status achieved by DXM conditioning could enhance the tolerogenic effect of short-term LPS-stimulated DCs, mice with CIA were treated with 2.0 mg/kg DXM for six days as described above, and at day 35, animals were split into three study groups, which received the following intraperitoneal injections: saline buffer (DXM group), DCs loaded with CII and stimulated for 4 hours with LPS (DXM/4hLPS/CII/DCs group), or unloaded DCs stimulated for 4 hours with LPS (DXM/4hLPS/DCs group). Also, animals with CIA injected at day 35 only with 4hLPS/CII/DCs or with vehicle were used as controls. 

**Figure 2:**
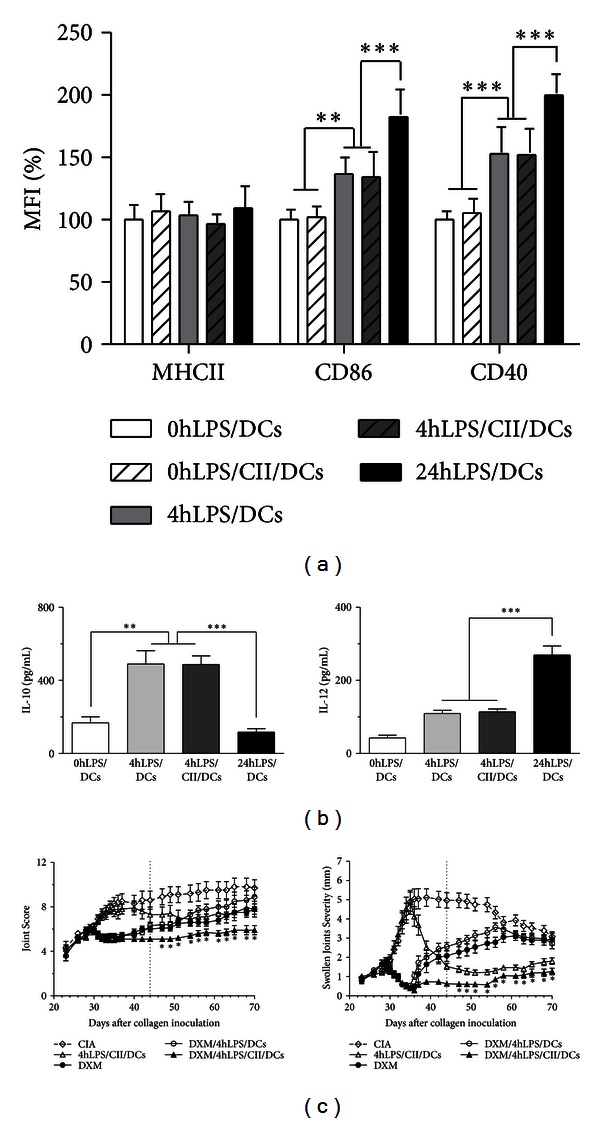
Dexamethasone (DXM) preconditioning improves the effect of 4-hour lipopolysaccharide-(LPS-) stimulated dendritic cells (DCs) in modulating active CIA. (a) Isolated CD11c+ DCs were stimulated with LPS for 4 hours (4hLPS/CII/DCs) and loaded for 24 hours with bovine type II collagen (CII) or left unloaded (4hLPS/DCs). LPS-untreated DCs loaded with CII (0hLPS/CII/DCs) or unloaded (0hLPS/DCs), or treated for 24 hours with LPS (24hLPS/DCs) were used as controls. The expression of major histocompatibility complex (MHC) class II and costimulatory molecules (CD86 and CD40) was analyzed by flow cytometry. Values are expressed as percentage of increase in mean fluorescence intensity (MFI) related to 0hLPS/DCs. Data from a representative experiment of three experiments performed are shown. **P < 0.01 and ***P < 0.001. (b) Cytokine production by differentially stimulated DCs was assessed by ELISA. Bars represent the mean of three experiments performed in duplicate. **P < 0.01 and ***P < 0.001. (c) Mice with active CIA received 2.0 mg/kg DXM from days 29 to day 34 after CIA induction (DXM group). Then, mice were inoculated intraperitoneally at day 35 with 5 × 10^5^ DCs as follows: 4-hour LPS-stimulated DCs (DXM/4hLPS/DCs) and 4-hour LPS-stimulated DCs loaded with CII (DXM/4hLPS/CII/DCs). The 4hLPS/CII/DCs group received 4-hour LPS-stimulated DCs loaded with CII, but without DXM preconditioning. The CIA control group corresponds to mice that did not receive any treatment. The two-tailed ANOVA test and Bonferroni's post-test were applied when comparing Joint Score and Swollen Joints Severity Score curves from day 44 to day 70. Data from a representative experiment of three experiments performed with 8–10 mice per group are shown. *P < 0.001 in DXM/4hLPS/CII/DCs versus CIA group.

 Previous to inoculation, DCs were phenotypically characterized by cell surface markers expression (MHC class II, CD86, and CD40) (Figure 2(a)) and functionally by evaluating their IL-10 and IL-12 production (Figure 2(b)). As depicted in Figure 2(a), 4hLPS/DCs and 4hLPS/CII/DCs displayed lower CD40 and CD86 expressions than 24hLPS/DCs (*P* < 0.001) and higher than 0hLPS/DCs or 0hLPS/CII/DCs (*P* < 0.001 and *P* < 0.01 for CD40 and CD86, resp.). As shown in Figure 2(b), we detected that both 4hLPS/DCs and 4hLPS/CII/DCs showed a higher IL-10 production than 24hLPS/DCs (*P* < 0.001) and 0hLPS/DCs (*P* < 0.01), while they produced lesser IL-12 than 24hLPS/DCs (*P* < 0.001). These data suggest that both phenotypic and functional features of 4hLPS/DCs are consistent with those displayed by tolerogenic DCs, as reported previously [6], and that these features are not affected by antigen loading.

 Once 4hLPS/DCs and 4hLPS/CII/DCs were characterized, they were administered to CIA mice, which had previously received the DXM pretreatment. We compared the severity curves from day 44 onwards to avoid the effect of DXM conditioning on its self. As depicted in Figure 2(c), according to the Joint Score, the DXM/4hLPS/CII/DCs group displayed a significantly less severe clinical disease from day 44 up to day 70, when compared to CIA mice (*P* < 0.001). In contrast, this effect was not observed in the 4hLPS/CII/DCs or DXM groups. This observation was also valid for the Swollen Joint Severity Score, although in this case both groups, DXM/4hLPS/CII/DCs and 4hLPS/CII/DCs, exhibit significantly lower scores than the CIA group (Figure 2(c)). Interestingly, the mice group receiving DXM/4hLPS/DCs showed a more severe clinical disease than mice from the DXM/4hLPS/CII/DCs or 4hLPS/CII/DCs groups, highlighting the antigen dependence of the tolerogenic effect.

### 3.3. Cytokine Production by CD4+ T Cells from CIA Mice Receiving DXM Preconditioning Followed by a Short-Term LPS-Stimulated DCs Injection

**Figure 3:**
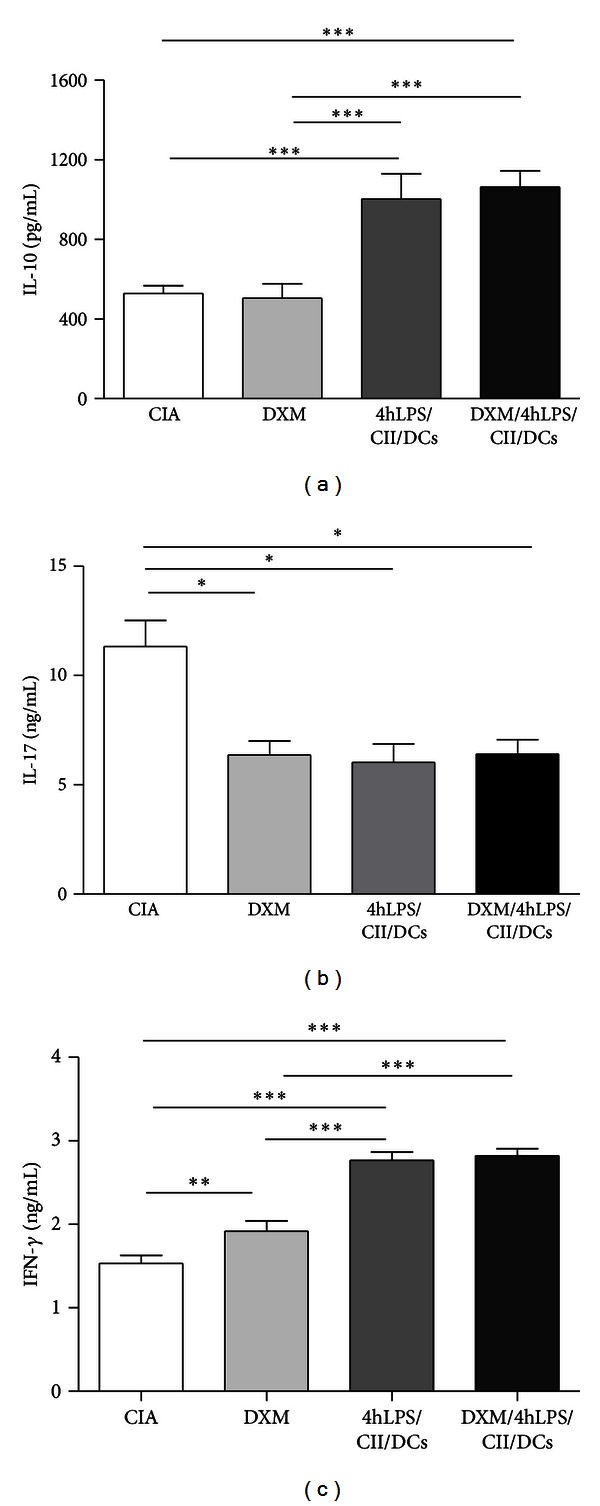
Cytokine production by CD4+ T cells from mice with active collagen-induced arthritis (CIA), treated with an inoculation of 4-hour lipopolysaccharide-(LPS-) stimulated dendritic cells (DCs) and dexamethasone (DXM) preconditioning. Mice received 2.0 mg/kg DXM from day 29 to day 34 after CIA induction (DXM group). Another group of mice were additionally injected at day 35 with 5 × 10^5^ 4-hour LPS-stimulated DCs loaded with type II collagen (CII) (DXM/4hLPS/CII/DCs group). The 4hLPS/CII/DCs group received 4-hour LPS-stimulated DCs loaded with CII, but without DXM preconditioning. The CIA control group corresponds to mice that did not receive any treatment. CD4+ T cells purified from mouse spleens at day 47 after CIA induction were stimulated with concanavalin A for 72 hours, and IL-10, IL-17, and IFN-*γ* levels in supernatants were quantified by ELISA. Bars represent the mean of three experiments.

 In order to evaluate whether the administration of 4hLPS/CII/DCs after preconditioning with DXM could modify the profile of cytokines secreted by CD4+ T cells in CIA mice, we assessed the secretion of IL-10, IL-17, and IFN-*γ* by stimulated splenic CD4+ T cells obtained at day 47 after CIA induction. We detected that CD4+ T cells from mice treated either with DXM/4hLPS/CII/DCs or 4hLPS/CII/DCs secrete higher IL-10 levels than those from the DXM group or from the CIA group (*P* < 0.001 for each comparison) (Figure 3). Interestingly, CD4+ T cells from all treated groups released significantly lower IL-17 levels when compared to those produced by CD4+ T cells from the CIA group (*P* < 0.05 for each comparison) (Figure 3). Furthermore, the evaluation of IFN-*γ* showed that CD4+ T cells from the 4hLPS/CII/DCs and DXM/4hLPS/CII/DCs groups released higher levels than those from the DXM group or the CIA group (*P* < 0.001 for each comparison) (Figure 3).

## 4. Discussion

Corticosteroids are potent anti-inflammatory drugs that are widely used in the treatment of rheumatoid arthritis (RA), having a beneficial role in both short-term and long-term management of the disease. In short-term use, corticosteroids are more effective anti-inflammatory agents than nonsteroidal anti-inflammatory drugs, and their long-term use has been shown to stop progression of bone erosions caused by RA, similar to other disease-modifying antirheumatic drugs [10].

Anti-inflammatory effects of corticosteroids have been associated with a strong inhibition of IL-2 by T cells, as well as signal transduction through the IL-2 receptor [11]. In addition, mice receiving DXM exhibit an expansion of regulatory T cells [12], and human CD4+ T cells treated *in vitro* with DXM increased the expression of regulatory T cell-associated transcription factor FoxP3 and the anti-inflammatory cytokine IL-10 [13], although it has been proposed that this induction is not correlated with an increased suppressive function [14]. 

Corticosteroids can affect other immune cells. For instance, it has been shown that DXM can induce an enhancement in human B cells ability to secrete IL-10 [15]. Also, it has been demonstrated that corticosteroids downregulate the production of IL-1*β*, IL-6, and TNF by monocytes and macrophages [16]. Studies in monocyte-derived DCs in humans and in bone marrow-derived DCs from mice have shown that hydrocortisone or DXM added *in vitro* strongly inhibit the production of IL-12p70, TNF, and IL-6 [17, 18]. Furthermore, hydrocortisone-treated monocyte-derived DCs induced less IFN-*γ* production and higher levels of IL-10 secretion by stimulated CD4+ T cells [17].

On the other hand, tolerogenic DCs have been used to restore tolerance in experimental autoimmune encephalomyelitis and CIA by preventive injections of TNF-maturated DCs [5, 7] or by therapeutic injection of LPS-stimulated DCs in mice with established CIA [6, 8]. Among others, studies performed by van Duivenvoorde et al., 2007, also demonstrate that a preventive inoculation with DXM-modulated DCs is able to inhibit the induction of CIA [7].

Based on the successful approach of nonmyeloablative conditioning previous to stem cell transplantation for the treatment of severe systemic autoimmune diseases, including experimental arthritis [19], we have hypothesized that the corticosteroid preconditioning of mice with established CIA may significantly reduce the inflammatory status, thereby strengthening the regulatory activity of tolerogenic 4hLPS/CII/DCs. Our results indicate that the conditioning with DXM was able to interfere with disease onset and progression during a short period. However, this quenching of the inflammation could have facilitated that antigen-pulsed tolerogenic DCs display a higher effectiveness in controlling CIA progression than that observed when tolerogenic DCs were inoculated without DXM conditioning.

Treatment with 4hLPS/CII/DCs alone or in combination with DXM significantly reduced the deleterious Th17 immune response, as we have previously reported [8]. In addition, both schemes were able to expand IL-10-producing CD4+ T cell subsets, which could be responsible for interfering with disease progression. Interestingly, the conditioning with DXM alone also inhibited the IL-17 production, providing a low inflammation environment, which could allow IL-10-producing CD4+ T cells to be generated by the effect of tolerogenic 4hLPS/CII/DCs, and to participate by inhibiting the autoimmune-mediated process in a synergic way. 

Remarkably, IFN-*γ* levels produced by CD4+ T cells increased significantly after 4hLPS/CII/DCs inoculation with or without DXM conditioning. As reported by van Duivenvoorde et al., 2007, and by our group [7, 8], tolerogenic DCs are able to induce a high percentage of IFN-*γ*-producing T cells in CIA mice. Our finding is in agreement with the protective role attributed to IFN-*γ*, according to the new paradigm accepted for CIA pathogenesis [20]; however, the present work was not intended to prove this issue.

Although the use of LPS with therapeutic purposes is inadmissible, it has been demonstrated that LPS activation is essential for inducing migratory and antigen-presenting activity in tolerogenic DCs [21]. The lack of proinflammatory stimulation does not allow DCs to upregulate polysialic acid production, a requirement to express high levels of the chemokine receptor CCR7, and they may therefore not be able to migrate to secondary lymphoid tissue [22]. Therefore, in order to improve the CCR7-mediated migratory capacity of tolerogenic DCs, without compromising their tolerogenic function, more investigation on the potential of safe LPS-derivatives or other pro-inflammatory reagents is required.

In conclusion, our findings support the use of DXM conditioning as an intervention that improves the effect of antigen-loaded short-term LPS-stimulated DCs as treatment for an animal model of RA. In addition, we demonstrated that CD4+ T cells from mice receiving the combined treatment produced high levels of IL-10 and IFN-*γ*, while they were low IL-17 producers. Nevertheless, regarding human application of this therapeutic strategy for RA, the antigenic proteins to be used for loading tolerogenic DCs is an important issue that remains unsolved and might be the target of intense research in the area for the future years.
